# Melatonin Suppresses the Expression of 45S Preribosomal RNA and Upstream Binding Factor and Enhances the Antitumor Activity of Puromycin in MDA-MB-231 Breast Cancer Cells

**DOI:** 10.1155/2013/879746

**Published:** 2013-04-07

**Authors:** Ji Hoon Jung, Eun Jung Sohn, Eun Ah Shin, Duckgue Lee, Bonglee Kim, Deok-Beom Jung, Ji-Hyun Kim, Miyong Yun, Hyo-Jeong Lee, Yong Koo Park, Sung-Hoon Kim

**Affiliations:** ^1^College of Oriental Medicine, Kyung Hee University, 1 Hoegi-dong, Dongdaemun-gu, Seoul 130-701, Republic of Korea; ^2^College of Medicine, Kyung Hee University, 1 Hoegi-dong, Dongdaemun-gu, Seoul 130-701, Republic of Korea

## Abstract

Since the dysregulation of ribosome biogenesis is closely associated with tumor progression, in the current study, the critical role of ribosome biogenesis related signaling was investigated in melatonin and/or puromycin induced apoptosis in MDA-MB-231 breast cancer cells. Despite its weak cytotoxicity, melatonin from 3 mM attenuated the expression of 45S pre-ribosomal RNA (pre-rRNA), UBF as a nucleolar transcription factor, and fibrillarin at mRNA level and consistently downregulated nucleolar proteins such as UBF and fibrillarin at protein level in MDA-MB-231 cells. Furthermore, immunofluorescence assay revealed that UBF was also degraded by melatonin in MDA-MB-231 cells. In contrast, melatonin attenuated the expression of survival genes such as Bcl-xL, Mcl-1, cyclinD1, and cyclin E, suppressed the phosphorylation of AKT, mTOR, and STAT3, and cleaved PARP and activated caspase 3 only at a high concentration of 12 mM. However, combined treatment of melatonin (3 mM) and puromycin (1 *μ*M) synergistically inhibited viability, attenuated the expression of 45S pre-rRNA and UBF, and consistently downregulated UBF, XPO1 and IPO7, procaspase 3, and Bcl-xL in MDA-MB 231 cells. Overall, these findings suggest that melatonin can be a cancer preventive agent by combination with puromycin via the inhibition of 45S pre-rRNA and UBF in MDA-MB 231 breast cancer cells.

## 1. Introduction

Breast cancers are one of the major casualties of death among women aged 30–55 years. Though several drugs such as tamoxifen, doxorubicin, and raloxifene have been clinically used to reduce the risk of breast cancer, side effects and resistance to chemotherapy are still hot issues [1]. Thus, the combined therapy of commercial anticancer agents with natural compounds is considered as an attractive strategy to reduce side effects or resistance, since it can meet the antitumor efficacy with lower doses of anticancer agents.

Melatonin, a hormone which is generated and secreted by the pineal gland during the night, is known to have various physiological functions by regulating sleep [2], depression [3], or age [4] and antioxidant activity [5]. Thereby, melatonin was reported to ameliorate several diseases such Alzheimer's disease [4], obesity [6], cardiovascular [7], bone disease [8], and cancer [9, 10] by combination or single treatment. Especially, melatonin in breast cancer was known to suppress the estrogen signaling pathway and vascular endothelial growth factor (VEGF)   [11] as well as inhibit aromatase expression and activity [12] in MCF-7 cells. Nevertheless, there is no evidence that ribosome biogenesis related signaling is involved in melatonin induced apoptosis in breast cancer cells until now. 

Ribosome biogenesis in the nucleolus is involved in cell growth and proliferation [13] via protein synthesis [14] and also involves the production of four rRNAs and approximately 80 ribosomal proteins. 45S pre-rRNA (pre-ribosomal RNA transcript) is transcribed by polymerase (Pol) I to generate mature 18S, 5.8S, and 28S rRNAs. Pol I is highly regulated in cancer [15] and overexpression of pre-rRNA synthesis is correlated with the rate of cancer cell proliferation [16, 17]. Thus, inhibition of rRNA synthesis makes cells undergo cell cycle arrest, apoptosis, or senescence [18]. Also, there is accumulating evidence that nucleolar proteins including upstream binding factor (UBF) recognize the promoter of ribosomal RNA genes and mediate the ribosomal RNA synthesis [19]. 

Thus, in the current study, the role of ribosome biogenesis was focused on apoptosis induced by melatonin and/or puromycin in MDA-MD-231 breast cancer cells. 

## 2. Material and Methods

### 2.1. Chemicals and Reagents

Melatonin (*N*-acetyl-5-methoxytryptamine; molecular weight: 232.28) and puromycin (C_22_H_29_N_7_O_5_, molecular weight: 471.51) were purchased from Sigma-Aldrich (Sigma, St. Louis, MO). Melatonin and puromycin were prepared in DMSO (Sigma, St. Louis, MO) and stored as small aliquots at −70°C for use. 

### 2.2. Cell Culture

Human breast cancer cell lines MDA-MB-231 (ATCC HTB-26) were obtained from American Type Culture Collection (ATCC) and cultured in RPMI1640 medium (Welgene, Daegu, South Korea) supplemented with 10% fetal bovine serum (FBS), 2 *μ*M L-glutamine and penicillin/streptomycin (Gibco, Carlsbad, CA, USA) at 5% CO_2_ conditions. 

### 2.3. Cytotoxicity Assay

To evaluate the cytotoxicity of melatonin and/or puromycin, MTT assay (Sigma, St. Louis, MO) was used. MDA-MB-231 cells were seeded onto 96-well microplates at a density of 2 × 10^4^ cells/well and treated with various concentrations of melatonin (1, 2, 4, 8, and 16 mM) and/or puromycin (0, 0.25, 0.5, 1, 2, 4 *μ*M) for 24 h. MTT solution (1 mg/mL) for 2 h was incubated and MTT lysis buffer (20% SDS and 50% dimethylformamide) was incubated overnight. Optical density (OD) was measured using a microplate reader (Molecular Devices Co., Sunnyvale, CA) at 570 nm. To calculate the viability of MDA-MB-231 cells, the percentage of viable cells in melatonin-treated group versus untreated control was used as the following equation. Cell viability (%) = [OD (drug) − OD (Blank)]/[OD (Control) − OD (Blank)] × 100.

### 2.4. Western Blotting

MDA-MB-231 breast cancer cells were lysed in Radioimmunoprecipitation Assay (RIPA) buffer (50 mM Tris-HCl, pH 7.4, 150 mM NaCl, 1% NP-40, 0.25% sodium deoxycholic acid, 1 M EDTA, 1 mM Na_3_VO_4_, 1 mM NaF and protease inhibitors cocktail). To quantify protein samples, Bio-Rad DC protein assay kit II (Bio-Rad, Hercules, CA) was used. Proteins were separated by electrophoresis on SDS-PAGE gel and electrotransferred onto a Hybond enhanced chemiluminescence (ECL) transfer membrane (Amersham Pharmacia, Piscataway, NJ). After blocking with 5% Bovine Serum Albumin (BSA), the membrane was probed with antibodies for fibrillarin, PARP, Bcl-x_L_, Mcl-1_L_, phospho-STAT3, phospho-mTOR (Cell signaling Technology, Danvers, MA), UBF, exportin 1 (XPO1), Importin (IPO7), caspase-3, cyclin D1, cyclin E, phospho-AKT (Santa Cruz Biotechnologies, Santa Cruz, CA), and *β*-actin (Sigma Aldrich Co., St. Louis, MO) followed by exposing to horseradish peroxidase (HRP) conjugated secondary anti-mouse or rabbit antibodies (AbD Serotec, Kidlington, UK). To detect protein expression, ECL system (Amersham Pharmacia, Piscataway, NJ) was used. 

### 2.5. Real-Time Quantitative RT-PCR (RT-qPCR)

Total RNA was isolated from MDA-MB-231 cells with QIAzol (Invitrogen). A reverse transcription kit (Promega,WI,USA) was used to construct the template cDNA. RT-qPCR was performed with the LightCycler TM instrument (Roche Applied Sciences, Indianapolis, IN) according to the manufacturer's protocol. The mRNA level of GAPDH was used to normalize the expression of genes of interest. The primers used were as follows: UBF forward: 5′-AAC ACC CAG ACT TCC CAA AG-3′, reverse: 5′-TCT CCG GAA GCT CCT TGT AT-3′; Fibrillarin forward: 5′-GGT GGA TGT GAT CTT TGC TG-3′, reverse: 5′-TGG CCT TAA TGG AAA TCA CA-3′; NCL forward: 5′-AGC AAA GAA GGT GGT CGT TT-3′, Reverse: 5′-CTT GCC AGG TGT GGT AAC TG-3′; 45S pre-rRNA forward: 5′-CTC CGT TAT GGT AGC GCT GC-3′, reverse: 5′-GCG GAA CCC TCG CTT CTC-3′; GAPDH forward: 5′-CCA CTC CTC CAC CTT TGA C-3′, reverse: 5′-ACC CTG TTG CTG TAG CCA-3′.

### 2.6. Immunocytochemistry Analysis

MDA-MB-231 cells in the presence or absence of melatonin were fixed in 4% paraformaldehyde, permeabilized in 0.3% Triton X-100, and blocked for 30 min in 1% BSA/10% fetal bovine serum at room temperature. Fixed cells were incubated with primary antibody (*α*-UBF) at 4°C overnight. After washed with PBS containing 0.1% BSA, the cells were incubated with Alex Fluor 568 goat rabbit-IgG antibody (Invitrogen) (1 : 500 dilution) at room temperature for 30 min. After washing twice, the cells were stained with 5 mg/mL 4,6-diamidino-2-phenylindole (DAPI; Sigma). Images were obtained by a Delta Vision imaging system (Applied Precision, Issaquah, WA, USA).

### 2.7. Statistical Analysis

Statistical analysis of the data was conducted using Sigmaplot version 12 software (Systat Software Inc., San Jose, CA) or Prism (GraphPad Software, Inc., CA, USA). All data were presented as means ± standard deviation (SD). The statistically significant differences between control and melatonin and/or puromycin treated cells were performed by the Student's *t* test.

## 3. Results 

**Figure 1:**
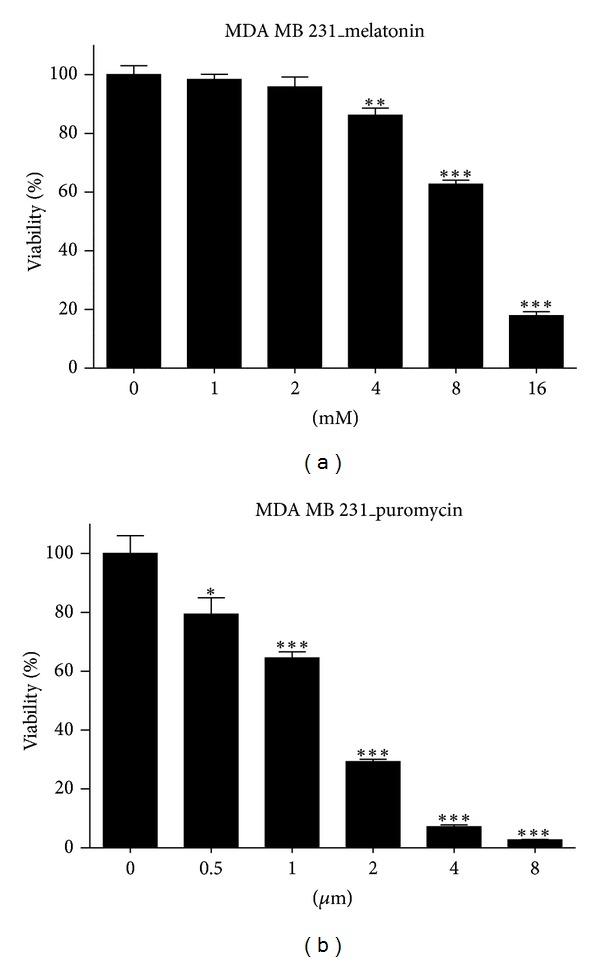
Cytotoxicity of melatonin and puromycin in MDA-MB-231 breast cancer cells. Various concentrations of melatonin (0, 1, 2, 4, 8, or 16 mM) (a) or puromycin (0, 0.5, 1, 2, 4, or 8 *μ*M/mL) (b) were treated in MDA-MB-231 cells for 24 h, and then cell viability was determined by MTT assay. The data were expressed as means ± SD. *P < 0.05, **P < 0.01, ***P < 0.001 versus untreated control.

**Figure 2:**
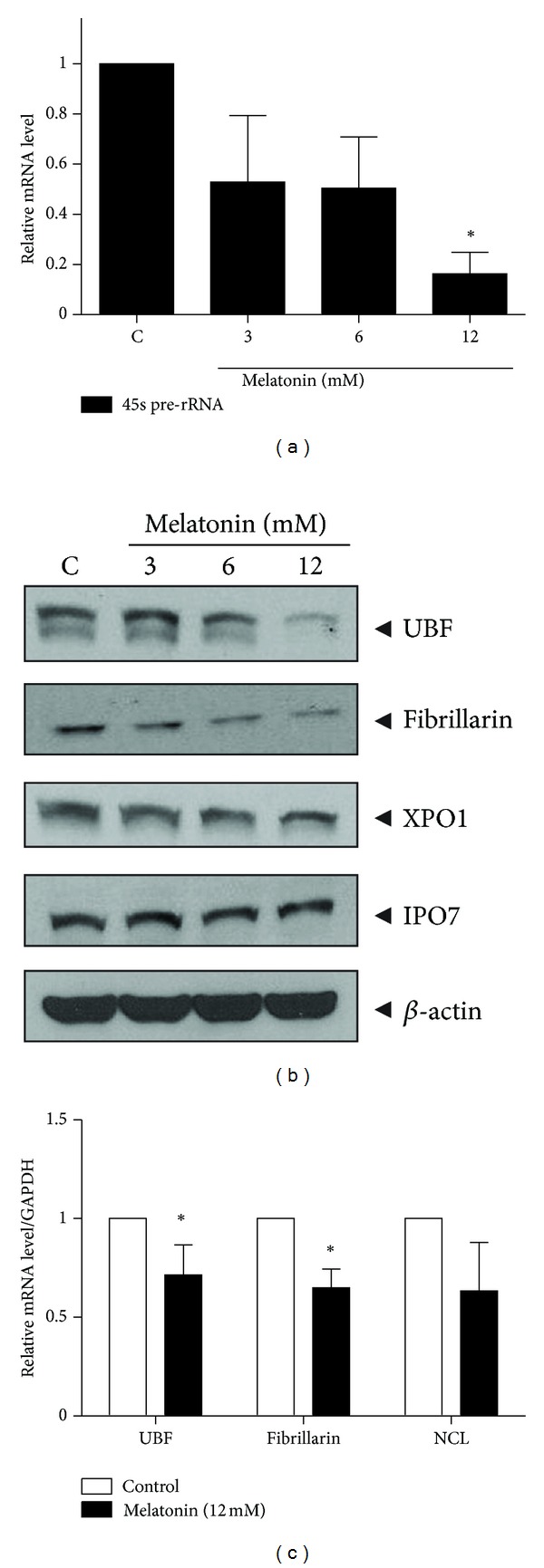
Effect of melatonin on the expression of 45S pre-rRNA and nucleolar proteins in MDA-MD-231 cells. (a) Effect of melatonin on 45S pre-rRNA. MDA-MB-231 cells were treated with various concentrations of melatonin (0, 3, 6, 12 mM) for 24 h. RNA was isolated and analysed for the expression of 45S pre-rRNA by RT-qPCR. (b) Effect of melatonin on nucleolar proteins by western blotting. Western blotting analysis was performed to detect UBF, fibrillarin, XPO1, IPO7, and *β*-actin. (c) Effect of melatonin on nucleolar proteins by RT-qPCR. RNA was isolated and analysed for the mRNA expression of UBF, fibrillarin, and NCL by RT-qPCR. GAPDH as internal control was used to normalize the expression of data. Data were presented as means ± S.D. *N* = 3, *P < 0.05 versus untreated control.

To target the synergistic antitumor activity by melatonin and puromycin, we evaluated the cytotoxicity of melatonin and/or puromycin in MDA-MB-231 cells by MTT assay. As shown in Figure 1, melatonin showed weak cytotoxicity only at the pharmacologically high concentrations of 8 and 16 mM in MDA-MB-231 cells, while puromycin exerted significant cytotoxicity in a dose dependent manner (0.5~4 *μ*g ≤ 1~8 *μ*M). To evaluate the potential of melatonin to regulate the ribosome synthesis, 45S pre-rRNA synthesis by RNA pol I in melatonin treated MDA-MB-231 cells was determined by RT-qPCR assay. As shown in Figure 2(a), though the inhibition was initiated from 3 mM in MDA-MB-231 cells, the statistical significance (*P* < 0.05) was recognized only at 12 mM of melatonin. 

Next, we assessed the effect of melatonin on the nucleolar proteins such as UBF, fibrillarin, or XPO1 and IPO7 which are involved in ribosome biogenesis. Our western blotting revealed that melatonin suppressed the expression of UBF and fibrillarin in a dose dependent manner but not IPO7 and XPO1 (Figure 2(b)). Consistently, UBF and fibrillarin at mRNA level were also downregulated in melatonin treated MDA-MB-231 cells, but not nucleolin (NCL) which is one of nucleolar proteins (Figure 2(c)). 

**Figure 3:**
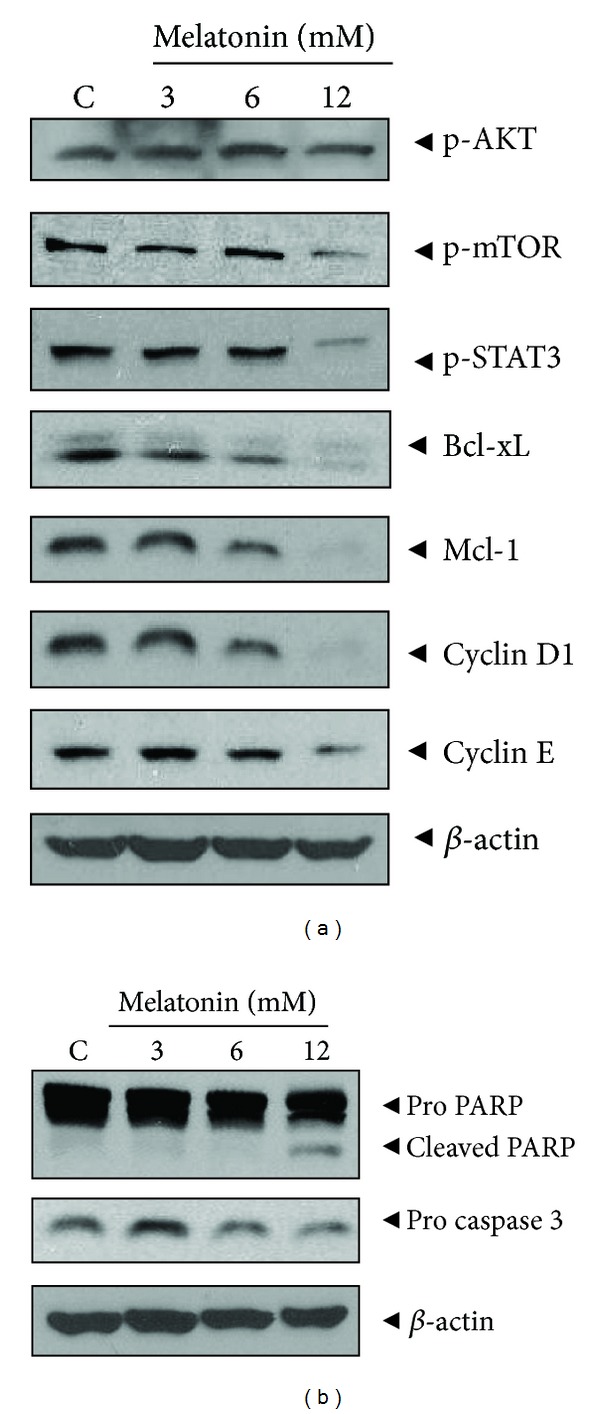
Effect of melatonin on the survival genes and apoptotic proteins in MDA-MB-231 cells. (a) Effect of melatonin on survival genes (b) Effect of melatonin on procaspase 3 and PARP. Cells were treated with melatonin (3 mM) for 24 h. Western blotting analysis was performed with antibodies of above survival, apoptotic genes, and *β*-actin.

We evaluated the effect of melatonin on the mTOR/AKT/STAT3 signals which are involved in cell growth. As shown in Figure 3, western blotting showed that melatonin down-regulated the phosphorylation of AKT, mTOR, and STAT3, attenuated the expression of survival genes such as McL-1, Bcl-xL, cyclin D1, and cyclin E, and activated caspase and cleaved PARP only at high concentration of 12 mM treated MDA-MB-231 cells.

**Figure 4:**
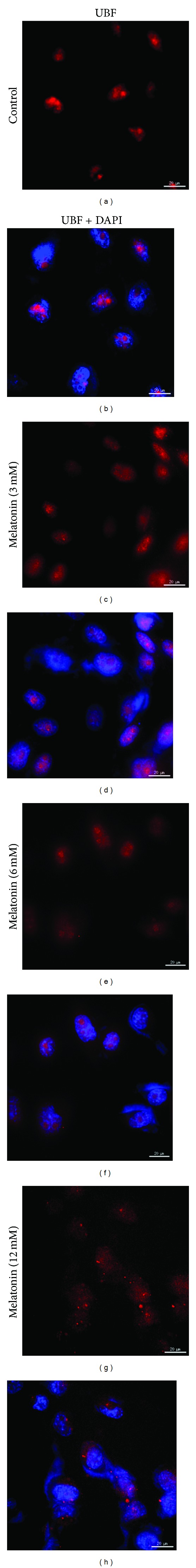
Distribution of UBF localization in melatonin treated MDA-MB-231 cells. Localization of UBF in MDA-MB-231 cells in the absence or presence of melatonin (3, 6, 12 mM) were fixed and immunostained with *α*-UBF. The localization of UBF was analyzed by a Delta Vision imaging system (Applied Precision). Nuclei were stained with DAPI. Scale bar, 20 *μ*m.

To check the effects of melatonin on intracellular localization of UBF, immunocytochemistry was performed. As shown in Figure 4, melatonin dispersed UBF aggregates at 3 or 6 mM from nucleoli to the nucleoplasm and proteolytically induced degradation at 12 mM in MDA-MB-231, while UBF was aggregated in the nucleoli in untreated control (Figure 4).

**Figure 5:**
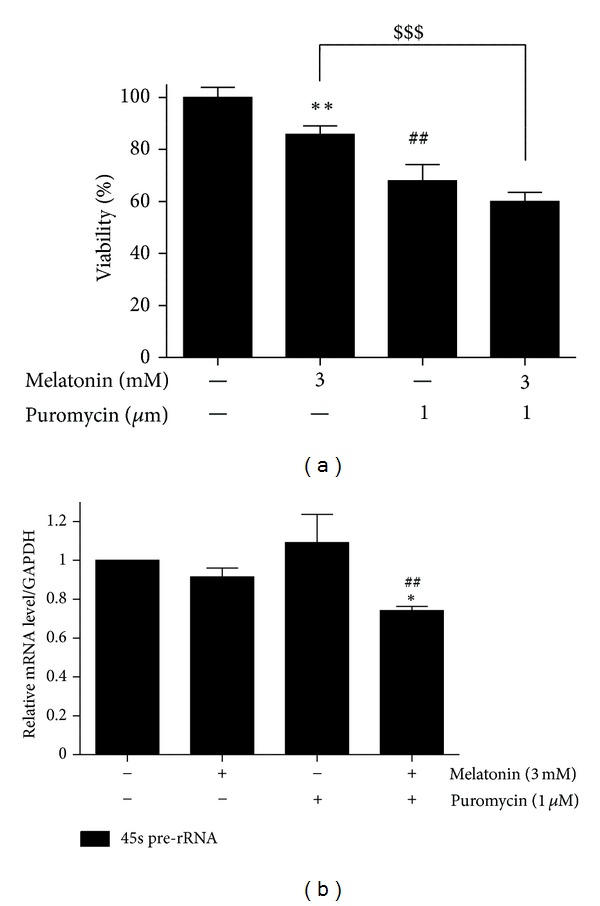
Combined effect of melatonin and puromycin on the viability and 45S pre-rRNA in MDA-MB-231 cells. (a) Effect of melatonin and/or puromycin on the viability of MDA-MB-231 cells. MDA-MB-231 cells were seeded at a density of 2 × 10^4^ cells per well on 96-well microplates and treated with melatonin (3 mM) and/or puromycin (1 *μ*m/mL) for 24 hr. Cell viability was assessed by MTT assay. Data were presented as means ± S.D.  ^##^ or **P < 0.01 versus untreated control, ^$$$^ *P* < 0.001 versus melatonin treated group. (b) Effect of melatonin and/or puromycin on 45S pre-rRNA. The mRNA expression of 45S pre-rRNA was determined by RT-qPCR. GAPDH was used to normalize the expression of pre-rRNA. Data were presented as means ± S.D. *P < 0.05 versus untreated control, ^##^ *P* < 0.01 versus puromycin treated group.

**Figure 6:**
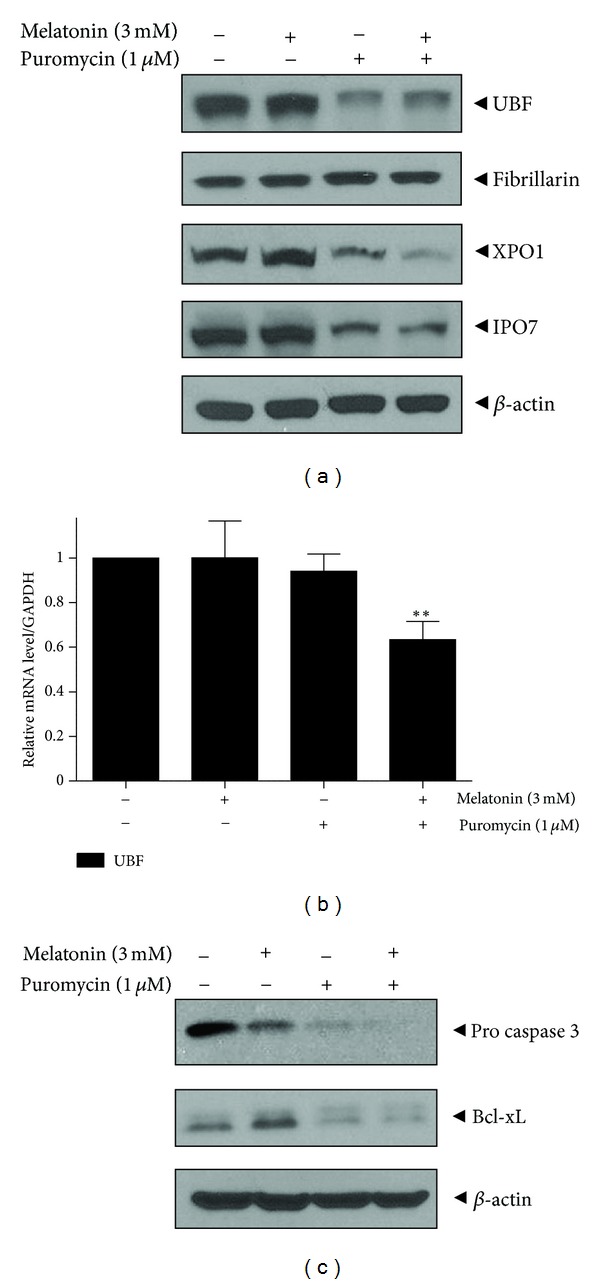
Combined effect of melatonin and puromycin on nucleolar protein and apoptosis related proteins rRNA in MDA-MB-231 cells. (a) Effect of melatonin and/or puromycin on nucleolar proteins by western blotting. (b) Effect of melatonin and/or puromycin on UBF at mRNA level. The mRNA expression of UBF was determined by RT-qPCR. GAPDH was used to normalize the expression of UBF. Data were presented as means ± S.D. **P < 0.01 versus untreated groups. (c) Effect of melatonin and/or puromycin on procaspase 3, BCL-xL by western blotting.

 To examine the synergistic effect of melatonin on puromycin mediated cell death, melatonin (3 mM) was cotreated with puromycin (1 *μ*M) in MDA-MB-231 for 24 h. Though combined treatment of melatonin and puromycin did not show any significant cytotoxicity against MDA-MB-231 cells (Figure 5(a)) compared to puromycin alone, cotreatment of melatonin and puromycin significantly suppressed 45S pre-rRNA in MDA-MB-231 cells compared to puromycin (Figure 5(b)). Next, to confirm whether ribosome biogenesis related genes mediate cytotoxicity of co-treatment of melatonin and puromycin in MDA-MD-231 cells, western blotting was carried out. As shown in Figure 6(a), UBF, XPO1, and IPO7 were suppressed by combined treatment of melatonin and puromycin, but not fibrillarin. Furthermore, RT-qPCR revealed that UBF expression at mRNA level was significantly downregulated by co-treatment of melatonin and puromycin in MDA-M231 cells (Figure 6(b)). In addition, the expression of procaspase 3 and BcL-xL was attenuated by cotreatment of melatonin and puromycin in MDA-MB-231 cells (Figure 6(c)).

## 4. Discussion

Ribosome biogenesis is a very complex process involving transcriptional and posttranscriptional steps in the nucleoli, where ribosomal RNA (rRNA) is synthesized into ribosomal subunits mainly through the RNA polymerase I (Pol I) transcription machinery [20]. Previous papers suggest that blocking or inhibiting rRNA synthesis can be a promising target for cancer treatment [21, 22]. Melatonin was reported to inhibit the growth of estrogen receptors (ER) positive breast cancer such as MCF-7, T47D and ZR-75-1 cells more effectively than estrogen negative breast cancer cells such as BT-20, MDA-MB-231, MDA-MB-364, Hs587t, T47Dco [23, 24]. Specifically, aggressive breast cancer cells displayed increased synthesis of 45S pre-rRNA with activation of an alternative pre-rRNA synthetic pathway [25]. UBF is an RNA polymerase I-specific transcription [26] and fibrillarin is involved in pre-rRNA processing, pre-rRNA methylation, and ribosome assembly [27]. In the current study, though melatonin showed weak cytotoxicity in MDA-MB-231 breast cancer cells, it effectively attenuated the expression of 45S pre-rRNA, UBF, and fibrillarin at mRNA level as well as downregulated nucleolar proteins such as UBF and fibrillarin at protein level in MDA-MB-231 cells. Consistently, immunofluorescence assay showed that UBF was dispersed or proteolytically degraded by melatonin, implying melatonin effectively disrupts ribosome biogenesis via inhibition of RNA polymerase I transcription.

There is accumulating evidence that survival genes are involved in ribosome biogenesis. Among them, mTOR coordinates transcription by all three classes of nuclear RNA polymerases [28, 29] and cyclin E upregulation promotes the growth and cell cycle progression by ribosomal protein L6 [30]. There are accumulating evidences that mTOR signal controls several steps in ribosome biogenesis such as the rRNA gene transcription and pre-rRNA processing [29]. Here, melatonin attenuated the expression of survival genes such as Bcl-xL, Mcl-1, cyclinD1, and cyclin E, suppressed the phosphorylation of STAT3, mTOR, and AKT and induced the cleavage of PARP and activated caspase 3 indicating that melatonin can induce apoptosis and inhibit the proliferation via inhibition of anti-apoptotic genes such as BCL-xL, Mcl-1, cyclin D1, cyclin E, p-STAT3, p-mTOR, and p-AKT at high concentration in estrogen negative MDA-MB-231 cells.

Belin et al. suggested that dysregulation of ribosome biogenesis has been linked with tumor progression by increasing ribosome synthesis in human breast cancer cells [25]. Ribosome synthesis in human breast cancer progression was increased up to 20% [25]. The relatively high level of ribosomal RNA (rRNA) was observed in rapidly proliferating tumor tissue [31] and rRNA synthesis also was linked to cellular proliferation rate. Thus, when rRNA synthesis becomes slow, cells undergo apoptosis, autophagy, or senescence [18]. Ribosomal RNA synthesis is a key molecular process for cell proliferation. In this process, UBF is involved in regulating rDNA transcription at the nucleolus, together with RNA polymerase I and recruiting the SL-1 and Pol I [32]. Fibrillarin mediates the processing of rRNA [33]. IPO7 and XPO1 for nuclear import and export signals regulate ribosomal stress [34]. In the present study, combined treatment of melatonin (3 mM) and puromycin (1 *μ*M) synergistically attenuated the expression of 45S pre-rRNA and UBF, suppressed cell viability, and also consistently abrogated the expression of UBF, XPO1, and IPO7, procaspase 3, and Bcl-xL in MDA-MB 231 cells, indicating the synergistic, antiproliferative and apoptotic effects between melatonin and puromycin even in estrogen negative MDA-MB-231 cells via inhibition of rRNA synthesis and Pol I transcription. These data were supported by previous similar evidence that melatonin promoted puromycin-induced apoptosis in human leukemia HL-60 cells [10]. 

In summary, melatonin inhibited 45S pre-rRNA in a dose dependent manner and attenuated the level of UBF and mTOR. Similarly, the pro-apoptotic signals were also up-regulated in melatonin treated MDA-MB-231 cells. Furthermore, co-treatment with melatonin and puromycin induced the apoptosis via downregulation of 45S pre-rRNA and UBF. Overall, these findings indicate that the inhibition of 45S pre-rRNA and UBF by melatonin is critically involved in the synergistic antitumor effect by combined treatment of melatonin and puromycin in aggressive MDA-MB 231 breast cancer cells and suggest melatonin has cancer chemotherapeutic potential in breast cancer cells.
