# Erratum to “Melatonin Suppresses the Expression of 45S Preribosomal RNA and Upstream Binding Factor and Enhances the Antitumor Activity of Puromycin in MDA-MB-231 Breast Cancer Cells”

**DOI:** 10.1155/2019/5741476

**Published:** 2019-07-21

**Authors:** Ji Hoon Jung, Eun Jung Sohn, Eun Ah Shin, Duckgue Lee, Bonglee Kim, Deok-Beom Jung, Ji-Hyun Kim, Miyong Yun, Hyo-Jeong Lee, Yong Koo Park, Sung-Hoon Kim

**Affiliations:** ^1^College of Oriental Medicine, Kyung Hee University, 1 Hoegi-dong, Dongdaemun-gu, Seoul 130-701, Republic of Korea; ^2^College of Medicine, Kyung Hee University, 1 Hoegi-dong, Dongdaemun-gu, Seoul 130-701, Republic of Korea

In the article titled “Melatonin Suppresses the Expression of 45S Preribosomal RNA and Upstream Binding Factor and Enhances the Antitumor Activity of Puromycin in MDA-MB-231 Breast Cancer Cells” [1], there was an error in Figure 3(a), where the fifth lane (Mcl-1) was mistakenly duplicated with the sixth lane (Cyclin D1), due to a production error. The correct figure is shown below.

## Figures and Tables

**Figure 3:**
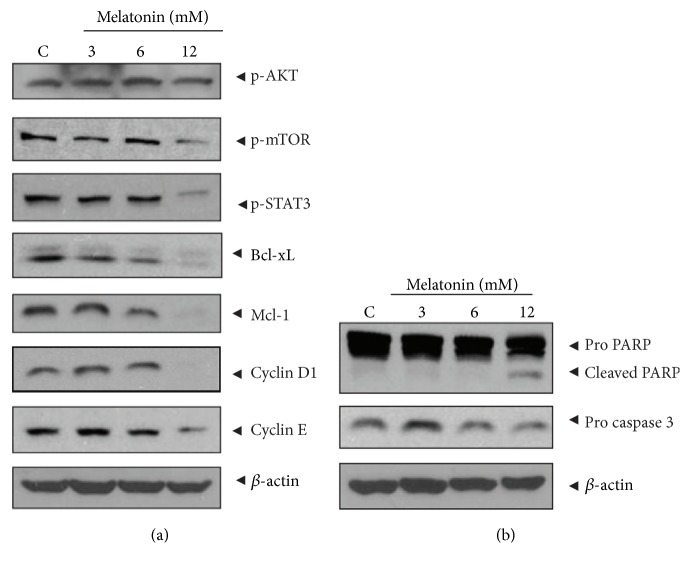
Effect of melatonin on the survival genes and apoptotic proteins in MDA-MB-231 cells. (a) Effect of melatonin on survival genes. (b) Effect of melatonin on procaspase 3 and PARP. Cells were treated with melatonin (3 mM) for 24 h. Western blotting analysis was performed with antibodies of above survival, apoptotic genes, and *β*-actin.
